# Life-saving emergency surgery due to delayed massive hemothorax 7 days after fall injury: a case report

**DOI:** 10.1186/s44215-023-00061-2

**Published:** 2023-08-21

**Authors:** Hideomi Ichinokawa, Takashi Sowa, Mikiko Suzuki, Kenji Suzuki

**Affiliations:** 1 Department of General Thoracic Surgery, Juntendo University Shizuoka Hospital, 1129, Nagaoka, Izunokuni, Shizuoka-Prefecture 410-2295 Japan; 2 Department of General Thoracic Surgery, Juntendo University Hospital, Tokyo, Japan

**Keywords:** Thoracic trauma, Delayed hemothorax, Emergency surgery, Vascular embolization, Intrathoracic angiography

## Abstract

**Background:**

Delayed hemothorax after thoracic trauma complicates approximately 7.4–36% of blunt traumas. Cases of delayed hemothorax that suddenly increase and require surgery are rare. We report a case of delayed massive hemothorax that was not relieved by vascular embolization but was successfully treated with surgery.

**Case presentation:**

The patient was a 45-year-old man. He was rushed to the emergency room after falling from the 4th floor, and he underwent emergency surgery. The patient was weaned off the ventilator on postoperative day (POD) 3 but had bleeding of 500 ml/h from his left chest drain on POD 7. We initially performed intrathoracic angiography for the bleeding. Bleeding from the 9th and 10th intercostal arteries was confirmed. Although vascular embolization was performed, 6 h later, 500 ml/h of bleeding was observed again from the drain, and emergency surgery was performed. We performed ligation of the left 9th and 10th intercostal arteries and intrathoracic hematoma removal. The patient was weaned off the ventilator 14 days after the second surgery and was transferred for rehabilitation on day 50.

**Conclusions:**

It is necessary to constantly monitor chest drainage and hemodynamics, especially within 7 days after injury, and to consider the possibility of emergency surgery.

## Background

The mortality rate of thoracic trauma is as high as 15.5% [1], with massive hemothorax being one of the causes of mortality. Massive hemothorax immediately after thoracic trauma is usually caused by injury to the intercostal artery, pulmonary laceration, great vessel injury, and diaphragmatic injury [2]. Delayed hemothorax is observed in approximately 7.4–36% of cases [3–5]. Many cases can be cured with thoracic drainage; however, cases of delayed massive hemothorax requiring surgery are extremely rare, with a reported incidence of 0.3% (5/1278) [6]. Herein, we report a case in which massive bleeding occurred on the 7th day after the injury, and chest computed tomography (CT) revealed rib deviation from the time of injury. The location of the bleeding was found using angiography, and hemostasis was achieved via surgery.

## Case presentation

**Fig. 1:**
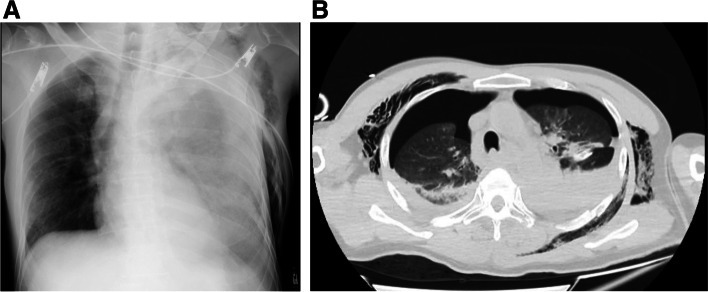
**A** A hemopneumothorax was found in the left thoracic cavity on admission chest radiography. **B** Chest computed tomography on admission revealed bilateral hemopneumothorax

**Fig. 2:**
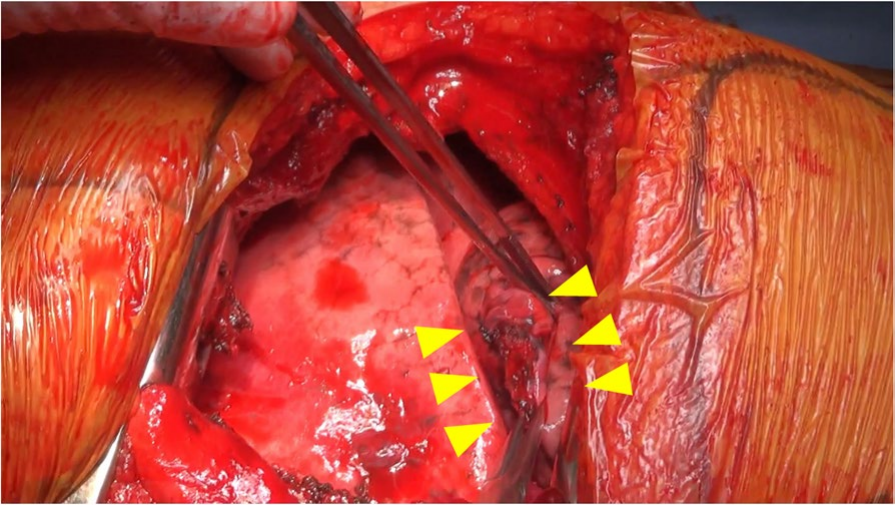
A laceration was found in the left upper lobe at the initial surgery (Yellow arrowhead: laceration site)

**Fig. 3:**
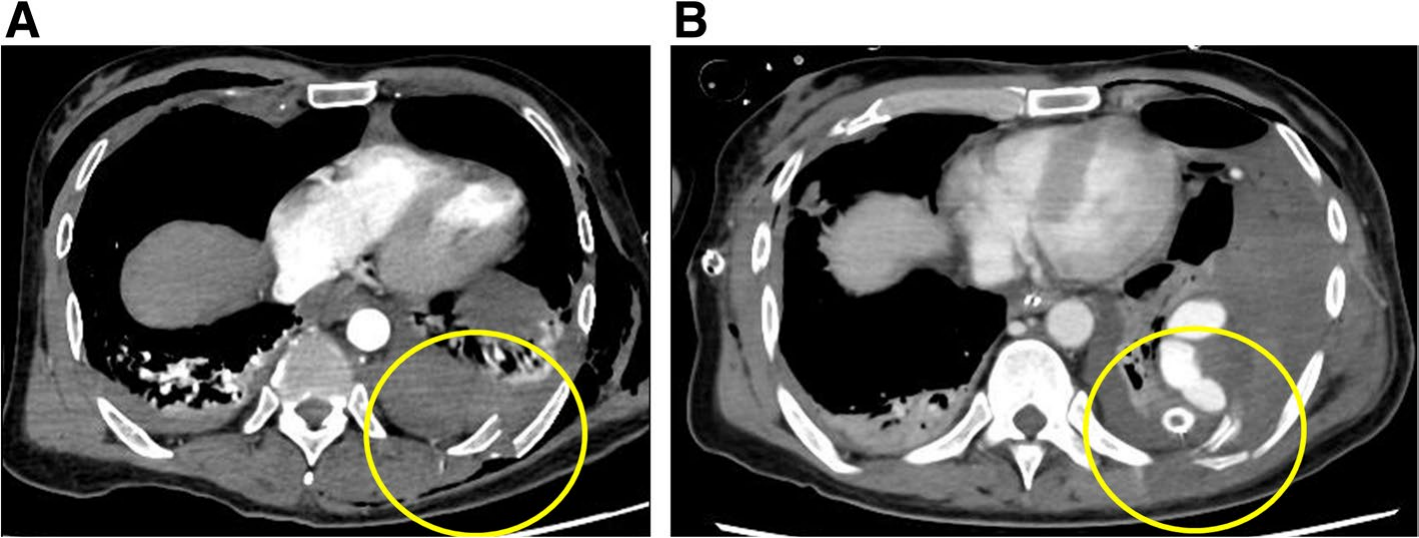
**A** Chest computed tomography before the initial surgery (yellow circle: 9th rib). **B** Chest computed tomography before the second surgery (yellow circle: 9th rib)

**Fig. 4:**
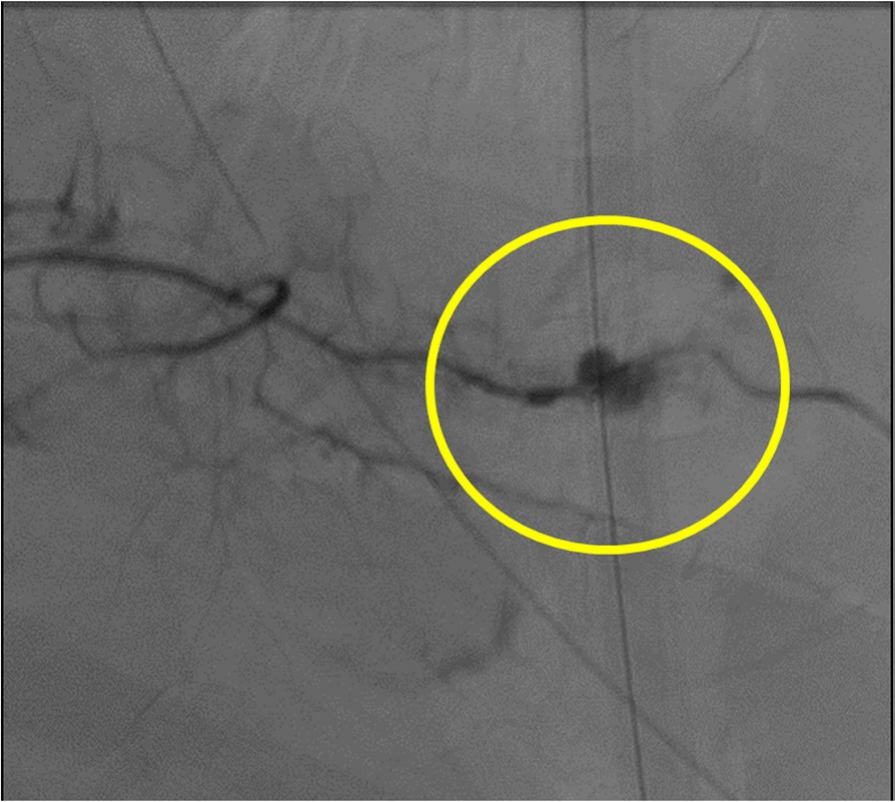
Outflow from the 9th intercostal artery on intrathoracic angiography (yellow circle: outflow location)

A 45-year-old man was brought to our hospital by ambulance because of injuries sustained in a fall from the 4th floor. Consciousness level on arrival was E1V2M2 on the Glasgow Coma Scale. Blood pressure was 70/52 mmHg, pulse was 112 beats/min, respiratory rate was 35 beats/min, and SpO2 was 93% (under the administration of a 15 L oxygen mask with reservoir). There was no history of antiplatelet drugs or anticoagulants. He underwent chest radiography and CT, which revealed bilateral hemopneumothorax (Fig. 1A, B). Bilateral chest drains were immediately inserted by the emergency department physician. A full-body examination revealed bilateral traumatic hemothorax, with left 4th to 10th rib fracture, right 5th to 7th rib fracture, bilateral intraventricular hemorrhage, Th6, Th12, and L2 vertebral body fractures, and right distal radius fracture. A bloody pleural effusion (1,000 ml was detected at the time of the insertion of the left drain, and another 1,000 ml of bloody pleural effusion was detected within the next hour. As such, we decided to conduct an emergency surgery. We performed a posterior lateral thoracotomy on the 5th intercostal space with a skin incision of 20 cm and found a laceration in the left upper lobe (Fig. 2) and bleeding from the 5th intercostal artery. Therefore, we sutured the left upper lobe and ligated the 5th intercostal artery. On postoperative day (POD) 3, we confirmed that he did not have a flail chest and that his breathing was stable; thus, he was weaned off the ventilator. After weaning from the ventilator, we considered the possibility of intrathoracic hemorrhage during body movement, so we monitored the progress without removing the chest drains on both sides. On POD 7, 400 ml/h of bloody drainage was observed from the left thoracic drain; his blood pressure was 80/40 mmHg, his heart rate was 140 beats/min, and he went into shock. Therefore, we suspected protracted bleeding in the thoracic cavity and performed contrast-enhanced chest CT. Leakage of the contrast medium was observed in the thoracic cavity, and deviation of the 9th rib into the thoracic cavity was confirmed compared with the chest CT at the time of the initial injury (Fig. 3A and B). Intrathoracic angiography was performed to confirm the outflow from the 9th to 10th intercostal artery (Fig. 4). Transarterial embolization was performed on the 9th and 10th intercostal arteries; however, bleeding from the thoracic drain continued, and a second emergency surgery was planned. We discovered a large hematoma in the left thoracic cavity, which was removed as much as possible. Bleeding from the 9th and 10th intercostal arteries was observed; therefore, the arteries were ligated. Intraoperative bleeding was 2560 ml, and 4U of red cell concentrate, 20 U of platelets, and 4 units of fresh frozen plasma were administered during the procedure. The patient was weaned off the ventilator on day 14 after the second surgery due to concerns about chest wall stabilization and rebleeding. He underwent a ventriculoperitoneal shunt for hydrocephalus on day 35 and was transferred to rehabilitation on day 50.

## Discussion and conclusions

Surgery for delayed massive hemothorax is rare in cases of thoracic trauma. Massive hemothorax is defined as blood drainage > 1500 ml after a closed thoracostomy or continuous bleeding at 200 ml/h for at least 4 h [6]. In the current case, when comparing the CT at the time of injury with that at the time of massive bleeding, we confirmed that there was a deviation of the posterior 9th rib. Therefore, we suspected damage to the descending aorta [7] and diaphragm [8] and bleeding from the intercostal artery due to the fracture segment. Gonzalez et al. reported that a posterior location and the displacement of at least one fractured rib on the initial CT are independent risk factors for delayed hemothorax [5]. Therefore, we believe that patients with posterior rib fractures due to trauma should be carefully monitored for sudden changes in their vital signs.

It is important to predict when a delayed hemothorax can occur. In the literature, it has been reported that the time of occurrence of delayed hemothorax ranged from 18 h to 44 days after the injury [3, 6, 9, 10]. We analyzed four papers [3, 6, 9, 10] and noted an incidence rate of 10% (7/70 cases) within 0–2 days, 79% (55/70 cases) within 3–7 days, and 11% (8/70 cases) 8 days after the injury. Consequently, we believe that patients should be monitored more carefully within 7 days (89%) after injury.

Second, we may also consider the stabilization of displaced ribs at the same time as the initial thoracotomy in some cases. The benefits include reducing the probability of delayed hemothorax and relieving severe pain caused by fractures. Disadvantages include longer operation time and increased invasiveness due to longer surgical wounds. This time, because it was an emergency operation, there was no anesthesiologist, and separate lung ventilation was not possible. In addition, the operation was not performed in an operating room but in an emergency center. Therefore, we chose not to stabilize the ribs in the initial surgery. Hemorrhagic shock is a life-threatening complication of trauma but remains a preventable cause of death. Early identification of the source of bleeding is critical for preventing adverse outcomes, including mortality. In the current case, angiography was useful in determining the location of the bleeding, but transarterial embolization was not effective in this case. According to American guidelines on the management of rapidly increasing hemothorax, bleeding > 1500 mL from a chest tube in any 24-h period, regardless of the mechanism, should prompt the consideration of surgical investigation [11]. Thoracotomy is the procedure of choice for the surgical examination of the chest when massive hemothorax or persistent bleeding is present.

In conclusion, we experienced a massive delayed hemothorax that occurred 7 days after a fall injury. We promptly performed chest CT and angiography, determined the cause of the bleeding, and relieved the patient with surgery. Delayed hemothorax should be suspected in patients with posterior rib fractures, and they should be carefully monitored within 7 days after injury.

## Data Availability

All data generated or analyzed during this study are included in this published article.

## Abbreviations

CT: Computed tomography
POD: Postoperative day
